# Investigation of Strand-Selective Interaction of SNA-Modified siRNA with AGO2-MID

**DOI:** 10.3390/ijms21155218

**Published:** 2020-07-23

**Authors:** Yukiko Kamiya, Yuuki Takeyama, Tomonari Mizuno, Fuminori Satoh, Hiroyuki Asanuma

**Affiliations:** Department of Biomolecular Engineering, Graduate School of Engineering, Nagoya University, Furo-cho, Chikusa-ku, Nagoya 464-8603, Japan; takeyama.yuuki@g.mbox.nagoya-u.ac.jp (Y.T.); mizuno.tomonari@b.mbox.nagoya-u.ac.jp (T.M.); sato.fuminori@f.mbox.nagoya-u.ac.jp (F.S.)

**Keywords:** siRNA, RNAi, AGO2, MID, serinol nucleic acid, off-target effect, guide strand selection, RNA, TEMPO, NMR

## Abstract

Small interfering RNA (siRNA) has been recognized as a powerful gene-silencing tool. For therapeutic application, chemical modification is often required to improve the properties of siRNA, including its nuclease resistance, activity, off-target effects, and tissue distribution. Careful siRNA guide strand selection in the RNA-induced silencing complex (RISC) is important to increase the RNA interference (RNAi) activity as well as to reduce off-target effects. The passenger strand-mediated off-target activity was previously reduced and on-target activity was enhanced by substitution with acyclic artificial nucleic acid, namely serinol nucleic acid (SNA). In the present study, the reduction of off-target activity caused by the passenger strand was investigated by modifying siRNAs with SNA. The interactions of SNA-substituted mononucleotides, dinucleotides, and (2,2,6,6-tetramethylpiperidin-1-yl)oxyl (TEMPO)-labeled double-stranded RNA (dsRNA) with the MID domain of the Argonaute 2 (AGO2) protein, which plays a pivotal role in strand selection by accommodation of the 5’-terminus of siRNA, were comprehensively analyzed. The obtained nuclear magnetic resonance (NMR) data revealed that AGO2-MID selectively bound to the guide strand of siRNA due to the inhibitory effect of the SNA backbone located at the 5’ end of the passenger strand.

## 1. Introduction

Small interfering RNAs (siRNAs) are duplexes of approximately 23 base pairs composed of passenger strands and guide strands. They promote gene silencing of messenger RNA (mRNA) targets complementary to the guide strand via RNA interference (RNAi). Notably, synthetic siRNAs have shown therapeutic potential. The first siRNA drug, namely patisiran, was approved by the Food and Drug Administration (FDA) in 2018 [1]. Chemical modifications of siRNAs can improve their resistance to nuclease digestion, increase potency, and reduce off-target effects [2,3].

Translational inhibition mediated by siRNAs is executed by the RNA-induced silencing complex (RISC), which comprises a siRNA guide strand and the Argonaute 2 (AGO2) protein. During the formation of the complex, the strand with the 5’ end, which is involved in the thermodynamically less stable region of base pairing, is selected for loading into RISC [4]. Strand selection is critical, as base pairing between the strand loaded into RISC and the mRNA target determine the selectivity of gene silencing. However, the control of the guide strand selection only by using RNA nucleotides is insufficient; therefore, it is necessary to develop methods that would result in a reduction of off-target activity mediated by the passenger strand-incorporated RISC. Various attempts have been made to reduce the activity of passenger strand-incorporated RISC, even via unintended formation of the complex, or alternatively, to improve the guide strand selectivity [2,3,5,6,7,8,9,10,11]. Nevertheless, further developments are required to achieve precise control.

**Figure 1:**
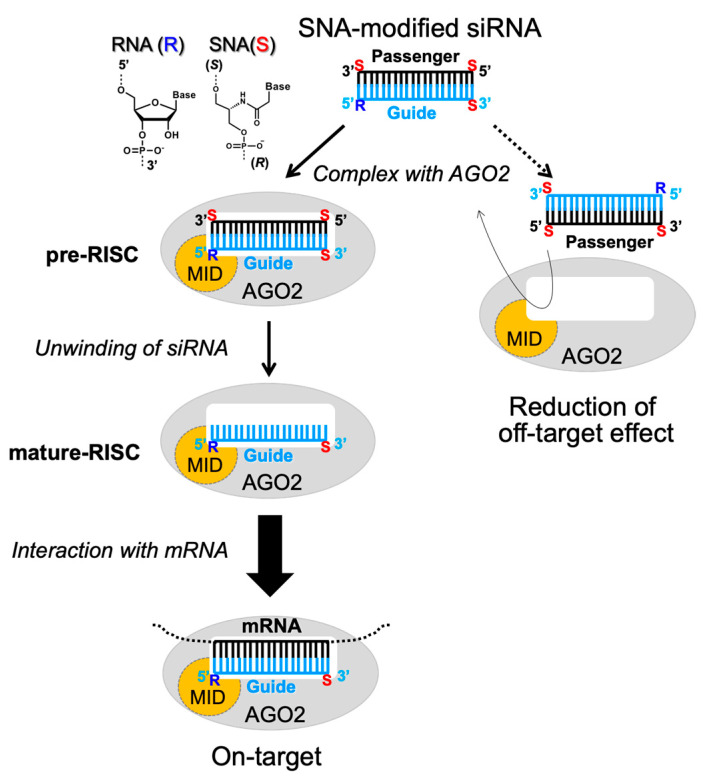
Schematic illustration of the proposed mechanism, by which serinol nucleic acid (SNA)-substituted small interfering RNA (siRNA) reduces off-target activity. In pre-RNA-induced silencing complex (pre-RISC), an interaction between the 5’ terminus of one strand and the MID domain results in selection of that strand for the formation of RISC. Off-target effects can occur if the passenger strand is selected. The presence of the SNA moiety at the 5’ terminus of the passenger strand inhibits the interaction with Argonaute 2 (AGO2)-MID, reducing off-target effects.

Structural studies revealed that AGO2 is composed of globular domains, i.e., N, PAZ, MID, and PIWI [12,13,14,15,16]. In the complex of micro RNA (miRNA) with full-length human AGO2, the 5’ end of miRNA is bound to the MID domain, while the 3’ end is bound to the PAZ domain. The seed region of miRNA is formed in an A-type helical geometry and is located in a narrow portion of the RNA binding groove of PIWI. Importantly, the 5’-terminal phosphate and nucleotide are not involved in pairing with the target mRNA. These moieties are located in a narrow binding pocket on the MID domain of AGO2 and exhibit stacking interactions and extensive hydrogen bonds with the amino acid residues of MID and PIWI. A previous study on the Y529E mutant showed that during the formation of RISC (pre-RISC), the 5’-end binding pocket of MID plays an important function in binding of miRNA to AGO2 [17]. Thus, to enhance the loading of the desired strand of siRNA into RISC, siRNAs exhibiting various 5’-end modifications have been proposed. For instance, introduction of metabolically stable (*E*)-5’-vinylphosphonate [18,19,20] or a 5’-triazol modifier, which was developed by computational screening [21,22], at the 5’ end of the guide strand increased the activity of siRNA. Moreover, preventing phosphorylation of the 5’-hydroxyl group of the passenger strand by Clp1 by 5’-*O*-methylation or 5’-morpholino modification reduced the formation of RISC with the passenger strand [23,24]. We previously reported an acyclic nucleic acid, specifically serinol nucleic acid (SNA), which stably hybridized with RNA [25,26,27]. In addition, we designed SNA-substituted siRNA, in which one residue at each terminus of the passenger strand and at the 3’ terminus of the guide strand was substituted with SNA (Figure 1) [28]. Consequently, the passenger strand-mediated off-target activity was effectively reduced and the on-target activity was enhanced by a simple SNA substitution without any additional modification of the 5’-hydroxyl group of siRNA. We expect that the SNA-substituted siRNA design can be applied in the development of nucleic acid drugs to knock down any disease-related genes and supplement miRNA. To make this design versatile, it is necessary to elucidate the mechanisms by which the SNA substitution at the terminal positions of siRNA reduces the off-target effects. We speculated that the SNA substitution inhibits the interaction of the passenger strand with AGO2-MID due to the considerable differences between the structures of SNA and ribose, resulting in loading of the guide strand into AGO2 (Figure 1). In the present study, we focused on the interactions between AGO2-MID and SNA-modified RNAs. Specifically, we performed isothermal calorimetry (ITC) and nuclear magnetic resonance (NMR) analyses to investigate the interactions between AGO2-MID and SNA-substituted mononucleotides, dinucleotides, and (2,2,6,6-tetramethylpiperidin-1-yl)oxyl (TEMPO)-labeled double-stranded RNAs (dsRNAs). We determined that AGO2-MID asymmetrically binds to the guide strand of the SNA-modified siRNA, in which the passenger strand contains an SNA moiety at the 5’ end.

## 2. Results and Discussion

### 2.1. Analyses of the Binding between AGO2-MID and Mono- and Dinucleotides

**Figure 2:**
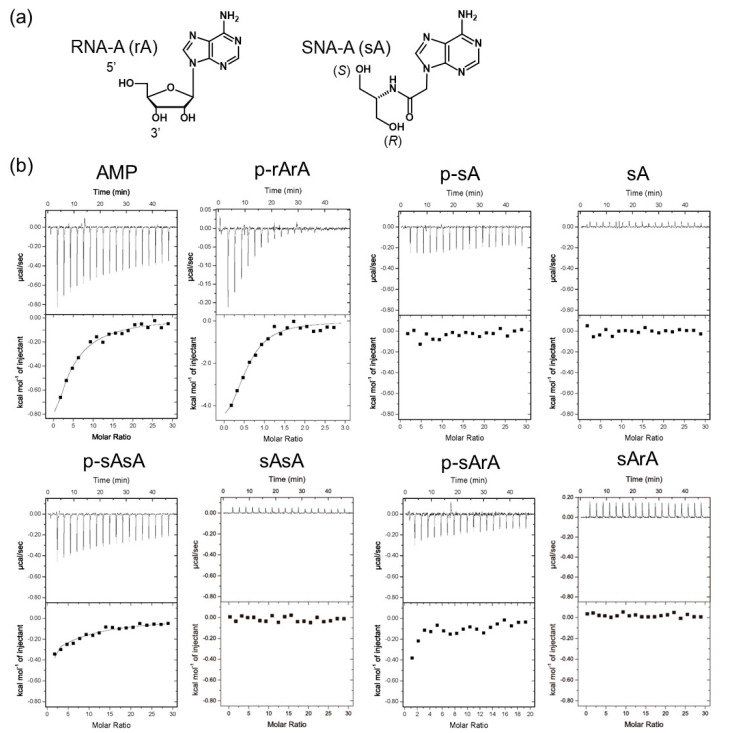
Isothermal calorimetry (ITC) analyses of the binding between AGO2-MID and mono- or dinucleotides. (**a**) Chemical structures of RNA and SNA mononucleotides. (**b**) ITC profiles of the binding between AGO2-MID and mono- and dinucleotides with or without terminal phosphate groups. ITC experiments were performed at 25 °C in 25 mM MES (pH 6.5), 200 mM NaCl, 1 mM tris(2-carboxyethyl)phosphine (TCEP). For each experiment, 20 μM AGO2-MID was loaded into the sample cell.

We first analyzed the binding affinity of AGO2-MID for mono- and dinucleotides using isothermal calorimetry (ITC). The affinity of AGO2-MID for riboadenosine (A), adenosine monophosphate (AMP), SNA-A (sA), and phosphorylated SNA-A (p-sA) was compared (Figure 2 and Figure S1 and Table 1). It was determined that although AMP bound to AGO2-MID (*K*_a_ 5.9 × 10^3^ M^−1^), p-sA did not. The RNA dimer containing a 5’-phosphate group (p-rArA) exhibited approximately 40 times higher affinity for AGO2-MID than AMP. The binding constant is within the range of typical protein-nucleotide interactions. Interestingly, the 5’-phosphorylated SNA-RNA dimer (p-sArA) was recognized by AGO2-MID (*K*_a_ 7.2 × 10^3^ M^−1^), although the binding affinity was considerably lower than that for the 5’-phosphorylated RNA dimer. The SNA dimer with an (*S*)-terminal phosphate group also bound to AGO2-MID (*K*_a_ 5.9 × 10^3^ M^−1^), indicating that the presence of the SNA moiety at the second residue from the 5’ end was tolerable for interaction with AGO2-MID. On the other hand, non-phosphorylated dimers of SNA or RNA did not detectably bind to AGO2-MID (Figure S1). These data suggested that the phosphate group and the ribose backbone of the 5’ terminal residue of siRNA cooperatively contributed to binding to AGO2-MID, which is consistent with previous reports [12,13,14,15,16]. Moreover, the results indicated that even the phosphorylated SNA residue did not bind to AGO2-MID.

**Figure 3:**
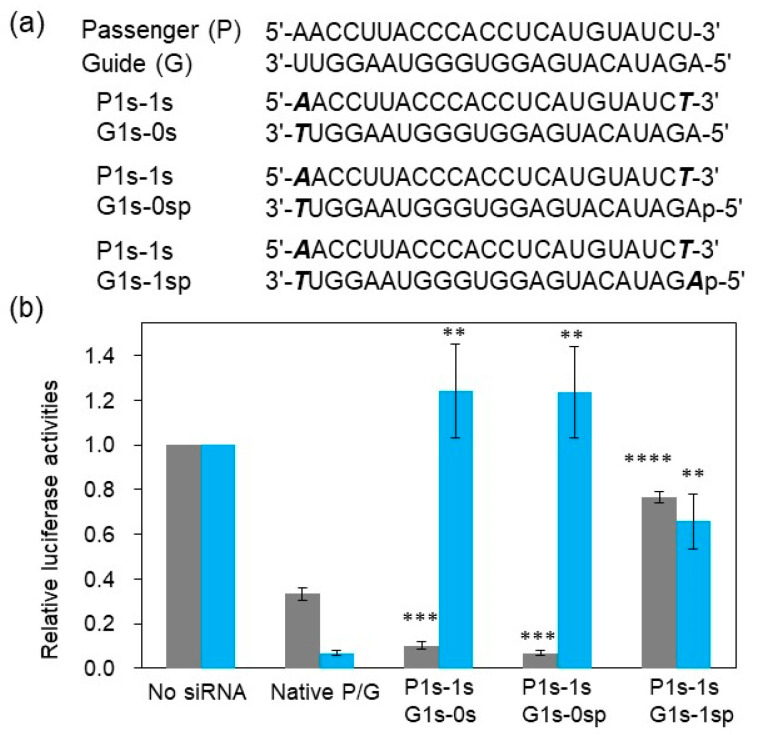
RNA interference (RNAi) activities of SNA-modified siRNAs. (**a**) siRNAs used in the luciferase assay. The upper strand is the passenger strand (P) and the lower strand is the guide strand (G). Bold, italic characters indicate SNA and p denotes 5’ phosphate. (**b**) RNAi activities of the guide strands (grey bars) and passenger strands (cyan bars) evaluated using reporter plasmids pGL3-On and pGL3-Off, respectively. Plotted are means and SD (*n* = 3) of *firefly*/Renilla luciferase signal relative to the untreated sample. **p < 0.01, ***p < 0.001, ****p < 0.0001 vs. native P/G.

We subsequently analyzed the RNAi activity and strand selectivity of SNA-modified siRNA. siRNAs modified with SNA at both 5’ and 3’ termini of the passenger strand and the 3’ terminus of the guide strand with and without a phosphate group at the 5’ end were prepared (P1s-1s/G1s-0s and P1s-1s/G1s-0sp, respectively; Figure 3). siRNAs modified with SNA at both termini of both strands and with a phosphate group at the 5’ end of the guide strand (P1s-1s/G1s-1sp) were also synthesized. Additionally, unmodified siRNA (native P/G) was prepared. A luciferase reporter assay was conducted to analyze the RNAi activities originating from the passenger strand and guide strand-incorporated RISC. In a previously reported assay, the pGL3-On and pGL3-Off plasmids, which were created by inserting the target sequence into the 3’-UTR of the firefly luciferase expression vector in the sense and antisense directions, respectively, were used to evaluate silencing induced by the guide and passenger strands [28]. Moreover, according to our previous study [28], both strands of the native P/G exhibited RNAi activity. Compared with unmodified siRNA, the guide strand of P1s-1s/G1s-0s, which contained an SNA residue at the 3’ end, but not the 5’ end, showed increased RNAi activity. In contrast, the passenger strand, in which both termini were modified with SNA, displayed reduced activity (Figure 3). Notably, P1s-1s/G1s-0sp, which had analogous SNA modifications to P1s-1s/G1s-0s and a 5’-phosphorylated guide strand, exhibited RNAi activity and guide strand selectivity equivalent to those of P1s-1s/G1s-0s (Figure 3).

The ITC results obtained herein as well as previously reported interaction analyses indicate that the presence of a phosphate group at the 5’ end on of the guide strand is critical for interactions with AGO2 [12,14,15]. We hypothesized that the guide strand of P1s-1s/G1s-0s was 5’phosphorylated by Clp1 in cells, which enhanced the incorporation into RISC [29] and resulted in activity equivalent to that of the chemically-phosphorylated siRNA. Moreover, for P1s-1s/G1s-1sp, both passenger and guide strands of siRNA were terminally modified with SNA. In this case, little silencing activity was observed, despite the presence of the 5’-phosphate group on the guide strand. This suggests that incorporation of siRNA into RISC was attenuated due to the SNA backbone on the 5’-terminal residue of the guide strand. These data imply that a 5’-terminal ribose backbone is prerequisite for accommodation of siRNA in the binding pocket of AGO2-MID.

### 2.2. TEMPO Modification of RNA for NMR Analyses

We then performed NMR analyses to obtain direct evidence of the strand selective interaction between dsRNA and AGO2-MID. To this end, the interaction between AGO2-MID and an RNA duplex modified with TEMPO was evaluated. It is known that an unpaired electron on the nitroxide group of TEMPO influences the peak intensity of NMR signals due to paramagnetic relaxation enhancement (PRE), which is related to r^−6^ [30]. Thus, TEMPO has been widely utilized in NMR analyses to provide long-distance information on the conformation and interactions of various biomolecules [31,32,33,34,35]. In this study, we attempted to evaluate the strand selectivity of AGO2-MID using a TEMPO-modified RNA duplexes.

**Figure 4:**
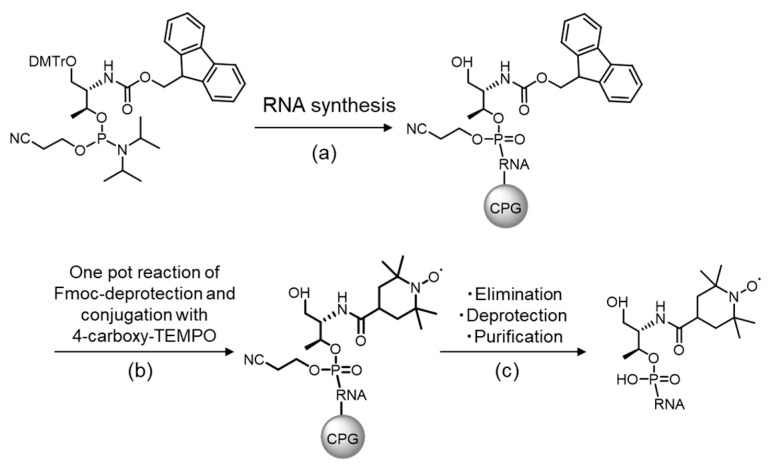
Scheme showing introduction of the (2,2,6,6-tetramethylpiperidin-1-yl)oxyl (TEMPO) moiety following solid-phase synthesis of RNA. (**a**) An Fmoc-protected D-threoninol was incorporated during solid-phase synthesis of RNA. (**b**) Deprotection of Fmoc and coupling of 4-carboxy-TEMPO was carried out simultaneously. RNA-conjugated controlled pore glass (CPG) was dispersed in dimethylformamide (DMF) and incubated with 4-carboxy-TEMPO (100 eq., TCI), 1-ethyl-3-(3-dimethylaminopropyl)carbodiimide (EDC) (250 eq.), hydroxybenzotriazole (HOBt) (250 eq.), and triethylamine (2.8 eq.) at 40 °C for 12–24 h. (**c**) The removal of RNA from CPG and deprotections were performed using conventional methods. The RNA was purified by reverse-phase high-performance liquid chromatography (HPLC).

The nitroxide radical on TEMPO is readily degraded during the detritylation and oxidation steps involved in the solid-support-based oligonucleotide synthesis [36,37]. TEMPO was previously effectively incorporated into RNA using optimized protocols of detritylation and oxidation in the presence of dichloroacetic acid and *tert*-butyl hydroperoxide, respectively [37]. Methods, in which the nitroxide group was incorporated at terminal positions of oligonucleotides for post-synthesis spin labeling of artificial ribose, nucleobase, or backbone moieties, have also been reported [38,39,40,41,42,43]. We initially attempted to prepare the TEMPO-modified oligonucleotide utilizing an amidite monomer of D-threoninol conjugated to TEMPO. Despite various modifications of the detritylation and oxidation steps during solid-phase synthesis, the nitroxide group was degraded after several cycles as a result of a reduction reaction (Figure S2a–c). Consequently, we introduced the TEMPO moiety in a post-synthetic manner. We first synthesized an amidite monomer of Fmoc-protected D-threoninol (D-thr-Fmoc) (Figure 4 and Scheme S1). Following conventional solid-phase oligonucleotide synthesis using D-thr-Fmoc, we conducted simultaneous deprotection of the Fmoc group and conjugation of TEMPO (Figure 4, step b). After the removal of RNA from controlled pore glass (CPG) and subsequent deprotection, the obtained RNAs were purified to yield TEMPO-modified RNA (Figure 4 and S2d).

### 2.3. Investigation of Strand Selection by AGO2-MID

The passenger strands were functionalized with TEMPO at either the 5’ or 3’ end and with an SNA residue at the opposite end. The obtained strands were hybridized to a guide strand modified with SNA at the 3’ end (P1s-Te/G1s-0s and PTe-1s/G1s-0s; Figure S3). To determine whether the TEMPO-modified siRNAs were able to induce gene silencing, we performed a luciferase reporter assay. Similar to the terminally SNA-modified siRNA P1s-1s/G1s-0s, P1s-Te/G1s-0s and PTe-1s/G1s-0s showed increased guide strand activity and significantly decreased passenger strand activity compared to the native siRNA (Figure S3).

**Figure 5:**
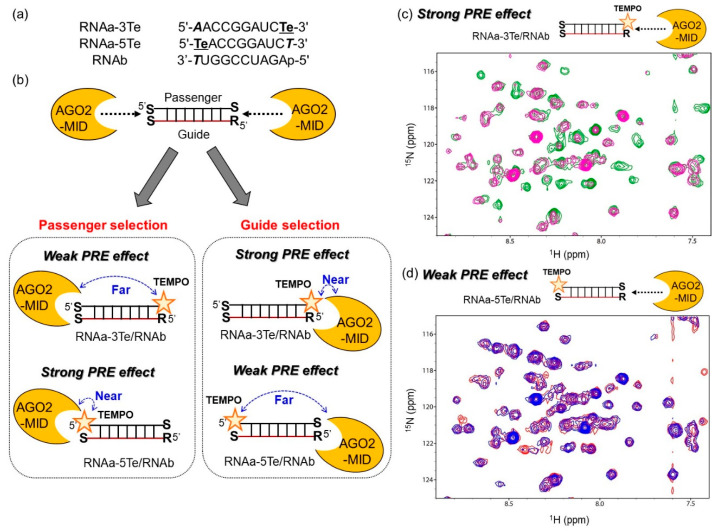
(**a**) RNAs used in the nuclear magnetic resonance (NMR) analyses. **Te** indicates TEMPO incorporated using a D-threoninol backbone. Bold, italic characters indicate SNA and p denotes the 5’-phosphate group. (**b**) Schematic illustration of the NMR analyses for investigation of strand selection by AGO2-MID. (c and d) ^1^H-^15^N heteronuclear single-quantum coherence (HSQC) NMR spectra of AGO2-MID (0.06 mM) with (**c**) RNAa-5Te/RNAb (0.4 mM) or (d) RNAa-3Te/RNAb (0.4 mM) in the absence (magenta or blue) and presence (green or red) of 2 mM ascorbic acid.

To analyze the interactions between SNA-modified siRNA mimics and AGO2-MID by NMR, we prepared two short TEMPO-modified 10-bp RNA duplexes with SNA modifications (Figure 5a). In one duplex, the passenger strand was modified with SNA at the 3’ end and TEMPO at the 5’ end (RNAa-5Te), while the guide strand was 5’-phosphorylated and modified with SNA at the 3’ end (RNAb). In the second duplex, the passenger strand was modified with SNA at the 5’ end and TEMPO at the 3’ end (RNAa-3Te), whereas the guide strand was RNAb. It is known that if AGO2-MID selectively interacts with the 5’ terminus of the guide strand, the NMR signals corresponding to AGO2-MID should be broadened upon the interaction with RNAa-3Te/RNAb due to strong PRE (Figure 5b). However, if AGO2-MID interacts with the passenger strand, the NMR signals of AGO2-MID should be broadened upon the interaction with RNAa-5Te/RNAb.

We first compared the ^1^H-^15^N heteronuclear single-quantum coherence (HSQC) spectra of AGO2-MID with and without TEMPO-labeled RNA duplexes in the presence of ascorbic acid, which reduced the nitroxide radical. Chemical shift perturbations of several NMR signals corresponding to the AGO2-MID residues were observed when the spectrum without RNA was compared to that with RNA (Figure S4). Notably, the perturbed chemical shifts were consistent with those previously reported for spectra of AGO2-MID with and without UMP [11,12,13]. We subsequently compared the spectra of AGO2-MID complexed with TEMPO-labeled RNA in the absence and presence of ascorbic acid. While NMR signals in the ^1^H-^15^N HSQC spectra of AGO2-MID in the presence of RNAa-3Te/RNAb were considerably broadened, much less broadening was observed in the presence of RNAa-5Te/RNAb (Figure 5c,d and S5). It is noteworthy that the broadened signals disappeared in the 2D-spectra. The acquired NMR data clearly indicated that the TEMPO moiety in RNAa-3Te was located in close proximity to AGO2-MID. This conclusion was based on the observation that some of the signals attributed to AGO2-MID in the 2D spectrum of RNAa-3Te/RNAb disappeared in the absence of ascorbic acid. Moreover, the disappearance of the signals was marginal in the spectra of RNAa-5Te/RNAb, in which the TEMPO moiety was away from AGO2-MID. Thus, the obtained results revealed that AGO2-MID preferentially interacted with the 5’ terminus of RNAb over the 5’ terminus of RNAa, the residue of which was composed of an acyclic backbone.

In conclusion, we successfully synthesized TEMPO-modified RNA, enabling NMR analyses, which revealed siRNA strand selectivity of AGO2-MID. Conjugation of TEMPO after solid-phase synthesis of RNA was necessary to prevent the reduction of the nitroxide moiety. A similar strategy could be used to post-synthetically introduce a TEMPO moiety at any desired position within an oligonucleotide. Moreover, other functional groups that would be unstable under conditions used for solid-phase oligonucleotide synthesis could be also introduced using this approach.

The conducted experiments demonstrated that modification of the 5’ end of the passenger strand of siRNA facilitates an asymmetric interaction between AGO2-MID and siRNA, significantly enhancing the selectivity for the desired guide strand. Based on the NMR and ITC data, we concluded that the SNA substitution at the 5’ end of the passenger strand considerably decreased the binding affinity of AGO2 for the passenger strand, resulting in selective incorporation of the guide strand into RISC. The outcomes of the present study provide valuable mechanistic insight into how the SNA modification at the 5’-terminus of the passenger strand of siRNA reduces off-target effects. The findings demonstrated herein provide a platform for further application of SNA modification in the design of therapeutic siRNAs.

## 3. Materials and Methods 

### 3.1. Syntheses of Dinucleotides and Oligonucleotides

The reagents for the synthesis of the phosphoramidite monomer of Fmoc-protected D-threoninol were purchased from Tokyo Kasei Co., Ltd. (Tokyo, Japan), Wako Pure Chemical Industries, Ltd. (Osaka, Japan), and Sigma-Aldrich, (Saint Louis, MO, USA). The reagents for the oligomer syntheses and Poly Pak II cartridges were obtained from Glen Research (Sterling, VA, USA) and ChemGenes (Wilmington, MA, USA). The columns for HPLC purification were purchased from Kanto Chemical Co., Ltd (Tokyo, Japan). The RNA oligonucleotides and oligonucleotides composed of SNA and RNA were acquired from Hokkaido System Science Co., Ltd. (Sapporo, Japan).

Phosphoramidite SNA monomers were synthesized according to a previously reported method [25,26]. Serinol-adenine (sA) was prepared by the deprotection of the 4,4′-dimethoxytrityl (DMT) group in DMT-serinol-adenine using trifluoroacetic acid. Dinucleotides (sAsA, rAsA, and sArA) were synthesized using SNA phosphoramidite and RNA monomers employing the ABI-3400 DNA synthesizer (Applied Biosystems, Waltham, MA, USA). Phosphorylation of sA and dinucleotides was conducted on solid-phase using phosphorylation reagent II (Glen Research, Sterling, VA, USA). D-Threoninol-Fmoc was introduced during solid-phase synthesis. For the deprotection of Fmoc and conjugation of TEMPO, RNA-conjugated CPG was dispersed in dimethylformamide (DMF) and incubated in the presence of 4-carboxy-TEMPO (100 eq., TCI), 1-ethyl-3-(3-dimethylaminopropyl)carbodiimide (EDC) (250 eq.), hydroxybenzotriazole (HOBt) (250 eq.), and triethylamine (2.8 eq.) at 40 °C for 12–24 h. During this step, some elimination of RNA from CPG was observed; therefore, the reaction time should not be exceeded. CPG was washed with DMF and CHCl_3_ and then dried. Subsequently, the RNA oligonucleotide was removed from CPG. The deprotection was conducted by incubating the oligonucleotide with a NH_4_OH/EtOH (3:1, v/v) solution at 55 °C for 8 h. After drying under vacuum, RNA was dissolved in 1 M tetra-*n*-butylammonium fluoride in tetrahydrofuran (THF) and stirred at 25 °C for 24 h for 2’ deprotection. The products were purified using diethylaminoethyl cellulose (DEAE) and reverse-phase high-performance liquid chromatography (HPLC) and analyzed by electrospray ionization mass spectrometry (ESI-MS) or matrix-assisted laser desorption/ionization-time-of-flight mass spectrometry (MALDI-TOF-MS) using the Autoflex II apparatus (Bruker Daltonics, Billerica, MA, USA).

### 3.2. ESI-MS Data 

p-sA Obsd. 345.085 (Calcd. for C_10_H_3_N_5_O_4_ 346.0791); sA Obsd. 265.115 (Calcd. for C_10_H_14_N_6_O_3_ 266.1127); p-rArA Obsd. 675.131 (Calcd. for C_20_H_26_N_10_O_13_P_2_ 676.4325); p-sArA Obsd. 674.143 (Calcd. for C_20_H_27_N_11_O_12_P_2_ 675.1316); p-sAsA Obsd. 673.1476 (Calcd. for C_20_H_27_N_12_O_11_P_2_ 674.1403); rArA Obsd. 595.165 (Calcd. for C_20_H_25_N_10_O_10_P 596.4538); sAsA Obsd. 593.196 (Calcd. for C_20_H_27_N_12_O_8_P 594.18124); sArA Obsd. 594.180 (Calcd. for C_20_H_26_N_11_O_10_P 595.1653).

### 3.3. MALDI-TOF-MS Data

P1s-Te: Obsd. *m/z* 7186.66 (Calcd. for P1s-Te: *m/z* 7187.08); PTe-1s: Obsd. *m/z* 7184.43 (Calcd. for PTe-1s: *m/z* 7178.08); RNAa-5Te: Obsd. *m/z* 3181.48 (Calcd. for RNAa-5Te: *m/z* 3175.60); RNAa-3Te: Obsd. *m/z* 3192.59 (Calcd. for RNAa-3Te: *m/z* 3185.94).

### 3.4. Protein Expression

DNA fragment encoding human AGO2-MID (432-578) was cloned between the BamHI and SalI sites of the pCold I plasmid (Takara, Kusatsu, Japan), in which the SUMO-TEV coding region was inserted between the NdeI and BamHI sites. Hexahistidine-tagged SUMO-TEV-hAGO2-MID was expressed in the *Escherichia coli* BL21(DE3)pLysS strain induced with 0.5 mM isopropyl β-D-thiogalactopyranoside. For NMR analyses, the protein was expressed in the M9 minimal medium with appropriate [^15^N] NH_4_Cl (Chembridge isotope laboratories Inc., Tewksbury, MA, USA)). The protein was purified using a Ni^2+^-NTA high-performance column (GE Healthcare, Chicago, IL, USA). The hexahistidine-modified SUMO was cleaved by incubation with proTEV (Promega, Madison, WI, USA) and further purified employing a Ni^2+^-NTA high-performance column and a cation exchange column (GE Healthcare, Chicago, IL, USA).

### 3.5. Cell Culture

The 293FT cells were cultured in Dulbecco’s modified Eagle’s medium supplemented with 10% fetal bovine serum, 80 µg/mL penicillin, and 90 µg/mL streptomycin. The cells were cultured at 37 °C with 5% CO_2_ in humidified air.

### 3.6. Dual Luciferase Assay

Reporter plasmids pGL3-On and pGL3-Off or pGlo-On and pGlo-Off were used for the dual luciferase assay [27,28]. The target sequence in pGL3-On and pGlo-On was 5’-AACCTTACCCACCTCATGTATCT-3’ and that in pGL3-Rv and pGlo-Off was the complementary sequence 5’-AGATACATGAGGTGGGTAAGGT-3’. The target sequences were inserted into the 3’-UTR region of the gene encoding firefly luciferase in pGL3. Co-transfections of the 293FT cells with siRNA (final concentrations 17 nM), 100 ng of pGL3 or pGlo, and 0.5 ng of the Renilla luciferase expression vector were performed using Lipofectamine 2000 (Invitrogen, Waltham, MA, USA) in 96-well plates according to the manufacturer’s instructions. Following incubation at 37 °C for 48 h, 75 μL of the medium was removed, and 75 μL of the Dual-Glo regent (Promega, Madison, WI, USA) was added. Firefly luciferase luminescence was measured on a Multi-label Plate Reader (EnSpire, Perkin Elmer, Waltham, MA, USA). Subsequently, 75 μL of the Dual-Glo Stop & Glo reagent was added, and the Renilla luciferase luminescence was measured.

### 3.7. Calorimetric Analysis 

The ITC experiments were performed at 25 °C using the iTC200 calorimeter (GE Healthcare, Chicago, IL, USA). AGO2-MID was dialyzed against 25 mM MES (pH 6.5), 200 mM NaCl, and 1 mM tris(2-carboxyethyl)phosphine (TCEP). 20 μM AGO2-MID was first loaded into the sample cell and then 2 μL of adenosine (3 mM), AMP (3 mM), sA (3 mM), p-sA (3 mM), rArA (3 mM), p-rArA (0.3 mM), sArA (3 mM), p-sArA (2 mM), rAsA (3 mM), sAsA (3 mM), or p-sAsA (3 mM) was added every 150 s.

### 3.8. NMR Analyses

AGO2-MID (0.06 mM) was mixed with TEMPO-labeled RNA duplex (0.4 mM) dissolved in 25 mM MES (pH 6.5), 100 mM NaCl, and 10% (*v*/*v*) D_2_O in the absence or presence of ascorbic acid (2 mM). The ^1^H-^15^N HSPQC spectra were acquired at 293 K using the Avance600 spectrometer equipped with a cryo-probe (Bruker BioSpin, Billerica, MA). The data were processed using the Topspin and Sparky software.

## Figures and Tables

**Table 1:** *K*_a_ values for the interaction of AGO2-MID with mono- and dinucleotides.

Mononucleot(s)ide	*K*_a_ (× 10^3^ M^−1^)	Dinucleotide	*K*_a_ (× 10^3^ M^−1^)
AMP	5.9	p-rArA	230
p-sA	n.d.	p-sArA	7.2
sA	n.d.	p-sAsA	5.9

AMP is adenosine monophosphate; p-sA is 5’ phosphorylated SNA adenosine; rA is adenosine. n.d. indicates that binding was not detected.

## Supplementary Materials

Supplementary materials can be found at https://www.mdpi.com/1422-0067/21/15/5218/s1. Figure S1: ITC analyses of binding of AGO2-MID with RNA mono and dinucleotides. Figure S2: MALDI-TOF-MS analyses of crude DNA or RNA oligomers containing D-threoninol-conjugated TEMPO. Figure S3: RNAi activities of TEMPO- and SNA-modified siRNAs. Figure S4: 1H-15N HSQC spectra of AGO2-MID complexed with RNA. Figure S5: 1H-15N HSQC spectra of AGO2-MID complexed with TEMPO-RNA in the absence or presence of ascorbic acid. Scheme S1: Synthesis of phosphoramidite monomer of Fmoc-conjugated D-threoninol.

## Abbreviations

siRNA	Small interfering RNA
AGO	Argonaute
RISC	RNA-induced silencing complex
NMR	nuclear magnetic resonance
TEMPO	2,2,6,6-tetramethylpiperidine 1-oxyl
SNA	Serinol nucleic acid
RISC	RNA-induced silencing complex
RNAi	RNA interference
AMP	Adenosine monophosphate
TCEP	Tris(2-carboxyethyl)phosphine hydrochloride
ITC	Isothermal titration calorimetry
PRE	paramagnetic relaxation enhancement
HSQC	heteronuclear single-quantum coherence
