# Supplementary material for: Investigation of Strand-Selective Interaction of SNA-Modified siRNA with AGO2-MID
Source: Int J Mol Sci. 2020 Jul 23;21(15):5218. doi: 10.3390/ijms21155218 (PMC7432901; doi:10.3390/ijms21155218)
Supplement: Supplementary file 1 [file ijms-21-05218-s001.zip › TEMPO-RNA_Supporting_proof_S1-S8.pdf]

Supplementary Materials

Investigation of strand-selective interaction of SNA-modified siRNA with AGO2-MID

Yukiko Kamiya,*[a] Yuuki Takeyama,[a] Tomonari Mizuno,[a] Fuminori Sato,[a] Hiroyuki Asanuma*[a]

[a]Department of Biomolecular Engineering, Graduate School of Engineering, Nagoya University, Furo-cho, Chikusa-ku, Nagoya 464-8603, Japan.

Table of Contents

1. Synthesis of Fmoc-conjugated D-threoninol monomer.... ....S2
2. Supplementary figures..S4
3. ^{1}H, ^{13}C, and ^{31}P NMR spectra of compounds...S9

1. Synthesis of phosphoramidite monomer of Fmoc-conjugated D-threoninol

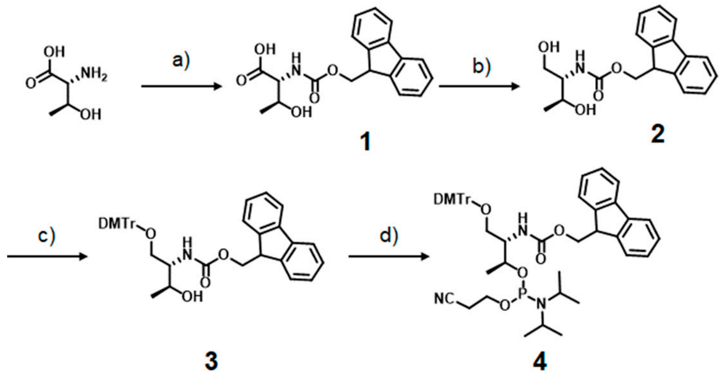

Scheme S1 Synthesis of phosphoramidite monomer of Fmoc-conjugated D-threoninol. a) 9-Fluorenylmethyl chloroformate, 1,4-dioxane, $NaHCO_3$, room temperature, overnight; b) DME, 4-methylmolphorine, ethyl chloroformate, room temperature, 30 min; c) 4,4'-dimethoxytrityl chloride, *N*,*N*-dimethyl-4-aminopyridine, *N*,*N*-diisopropylethylamine, pyridine, CH_2Cl_2, 0 °C, 30 min → room temperature, 1.5 h; d) 2-cyanoethyl *N*, *N*-diisopropylchlorophosphoramidite, Et_3N, CH_2Cl_2, 0 °C → room temperature, 20 min.

Synthesis of compound 2

D-Threonine (5.95 g, 50 mmol) was dissolved in saturated $NaHCO_3$ solution (100 ml). After dropwise addition of 9-fluorenylmethyl chloroformate dissolved in 1,4-dioxane (13.3 g, 50.2 mmol), the mixture was stirred overnight at room temperature. The solvent was reduced by evaporation, and the aqueous layer was extracted twice with diethyl ether, and the pH was adjusted to between 1 and 2 using concentrated H_2SO_4. The organic layer was extracted with ethyl acetate. The mixture was washed with a saturated solution of NaCl. After drying over $MgSO_4$, the solvent was removed by evaporation to afford compound **1** (yield 99 %). Compound **1** (17.0 g, 49.7 mmol) was dissolved in dry DME (50 ml) under nitrogen. The mixture was cooled on dry ice-methanol, and then 4-methylmolphorine (5.37 ml, 49.7 mmol) and ethyl chloroformate (4.73 ml, 49.7 mmol) were added. After stirring for 10 min on dry ice-methanol, the mixture was further stirred for 30 min at room temperature. Then mixture was then added to $NaBH_4$ (2.82 g, 74.6 mmol) dissolved in H_2O (100 ml) on ice. The solvent was reduced by evaporation, and the organic layer was collected with ethyl acetate. The mixture was washed with H_2O, with a saturated aqueous solution of $NaHCO_3$, and with a saturated solution of NaCl. After drying over $MgSO_4$, the solvent was removed by evaporation. The crude product (compound **2**) was used in the next step without further

purification (quantitative yield).

Synthesis of compound 3

Dry pyridine (80 mL) solution containing compound **2** (13.0 g, 39.8 mmol) and *N,N*-diisopropylethylamine (10.1 mL, 59.7 mmol) was cooled on ice under nitrogen. 4,4'-Dimethoxytrityl chloride (14.8 g, 43.8 mmol) and *N,N*-dimethyl-4-aminopyridine (0.61 g, 5.0 mmol) dissolved in 80 mL of dry CH_2Cl_2 were added to the mixture. The reaction mixture was stirred for 30 min on ice and then for 1.5 h at room temperature. The reaction was quenched by addition of MeOH (10 ml). The solvent was dried under vacuum. The mixture was dissolved with ethyl acetate and washed with a saturated aqueous solution of $NaHCO_3$ and then with a saturated solution of NaCl. The solvent was reduced under vacuum, and purification by silica gel column chromatography (hexanes/AcOEt (2:1, v/v), 3% Et_3N) afforded compound **3** (29.9 mmol, yield 75%).

^{1}H NMR [500 MHz, $CDCl_3$]: δ = 7.78-6.79 (m, aromatic protons of DMT and Fmoc), 4.45 and 4.35 (m, 2H, -COO-C*H2*), 4.25 (m, 1H, proton of Fmoc), 4.1 (m, 1H, -C*H*(CH_3)OH), 3.76 and 3.75 (s, 6H, -OC*H*$_3$), 3.68 (m, 1H, -C*H*-NHCO-), 3.45 and 3.25 (m, 2H, -C*H*$_2$-ODMT), 1.17 (d, 3H, -CH(OH)C*H*$_3$). ^{13}C-NMR [$CDCl_3$, 125 MHz] δ = 159.0, 143.6, 141.1, 135.1, 129.5, 127.4, 126.6, 124.6, 119.6, 112.9, 101.3, 76.2, 67.9, 66.7, 65.1, 54.9, 47.0, 19.4. HRMS calcd for $C_{40}H_{39}NO_6$ 629.27774; found 629.2783 (M^+).

Synthesis of compound 4

Et_3N (1.1 mL) and 2-cyanoethyl *N,N*-diisopropylchlorophosphoramidite (1.0 mL) were added to a solution of compound **3** (1.95 g, 3.0 mmol) in dry CH_2Cl_2 (10 mL) on ice, and the solution was stirred for 15 min at room temperature. Purification by silica gel column chromatography (hexanes:ethyl acetate (3:1 to 2:1, v/v), 2% triethylamine) afforded compound **3** (1.69 g, 2.04 mmol, yield 65.8%).

^{1}H-NMR [500 MHz, $CDCl_3$]: δ = 7.8-7.13 (m, aromatic protons of Fmoc), 7.33-6.70(m, aromatic protons of DMT), 4.52- 4.08 (m, -CO-O-C*H*2-Fmoc, Fmoc, and -C*H*(CH_3)O-P), 4.96-3.82 (2H, -C*H*$_2$-CH_3-CN), 3.79-3.70 (6H, -O-C*H*3), 3.58-3.43 (1H, -C*H*-CH_2-O-), 3.58-3.09 (2H, -CH-C*H*$_2$-O-), 2.61-2.53 (2H, -N-C*H*-CH4), 2.40-2.33 (3H, -C*H*$_3$-CN), 1.29-1.03 (m, 15H, CH(C*H*$_3$)$_2$). ^{13}C-NMR [$CDCl_3$, 125 MHz] δ =158.3, 156.2, 144.2, 143.6, 141.1, 135.7, 129.8, 127.3, 126.6, 124.9, 119.7, 112.8, 85.9, 76.4, 66.4, 63.2, 62.8, 57.9, 47.1, 42.8, 24.0, 20.0, 19.2. ^{31}P NMR [202 MHz, $CDCl_3$]: δ =148.05, 148.32. HRMS calcd for $C_{49}H_{56}N_3O_7P$ 829.38559; found 830.3907 ($M+H^+$).

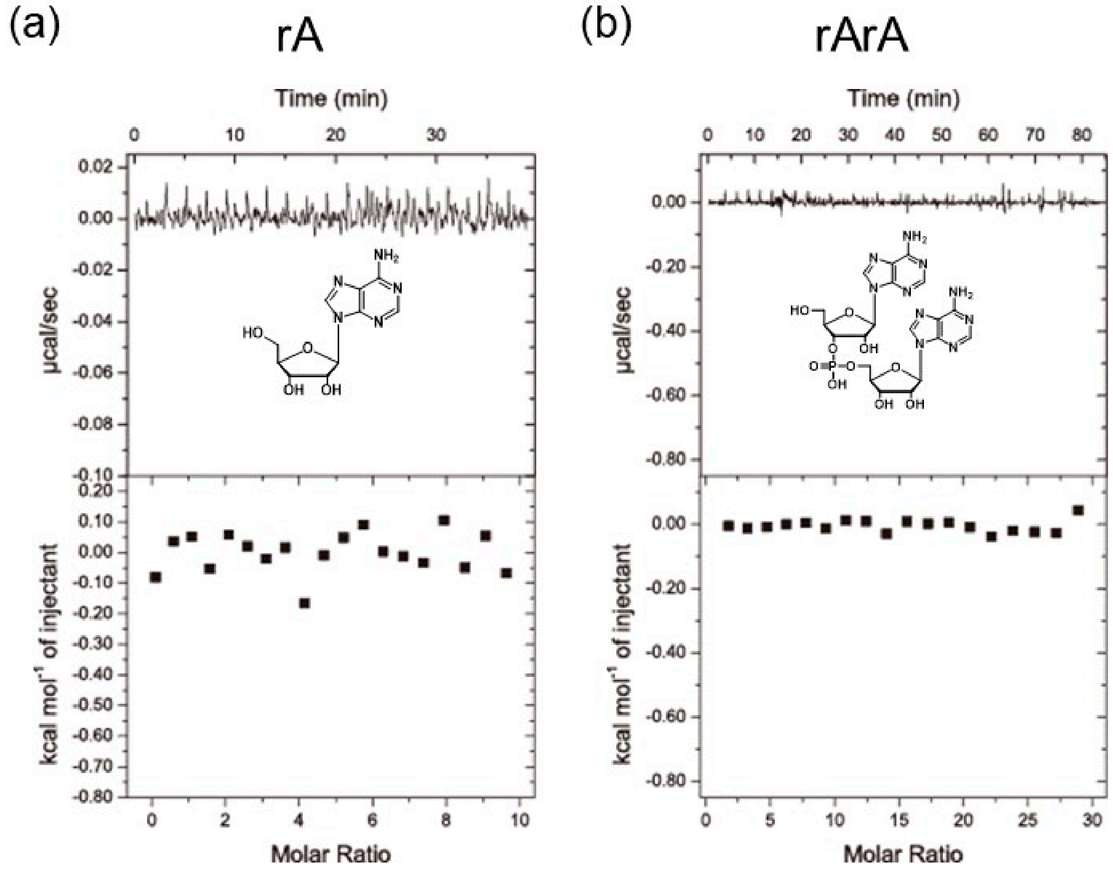

Figure S1 ITC analyses of binding of AGO2-MID with RNA mono and dinucleotides.

ITC profiles of the binding of AGO2-MID with rA (a) and rArA (b) without terminal phosphate groups. ITC experiments were performed at 25 °C in 25 mM MES (pH 6.5), 200 mM NaCl, 1 mM TCEP. For each experiment, 20 μM AGO2-MID was loaded into the sample cell.

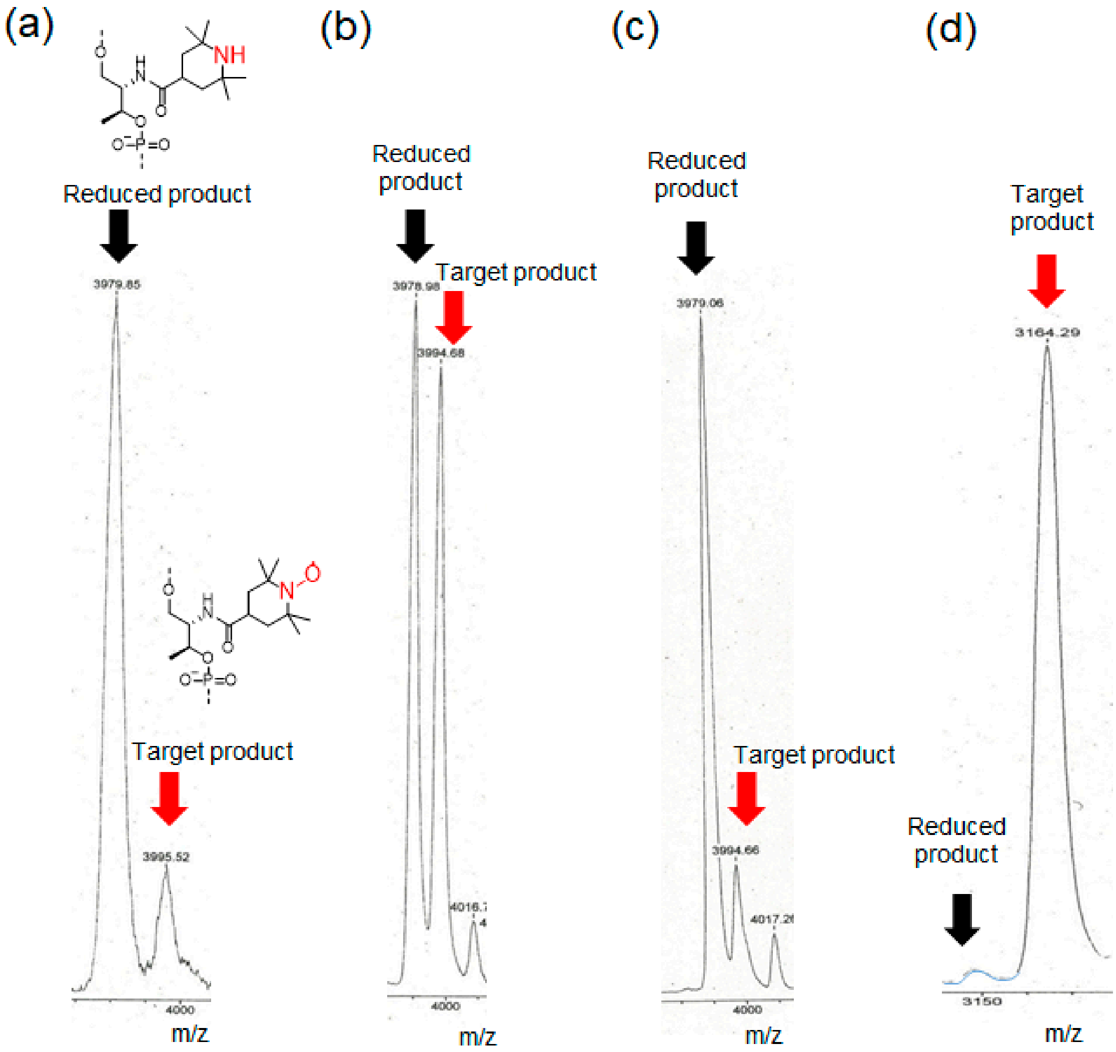

Figure S2 MALDI-TOF-MS analyses of crude DNA or RNA oligomers containing D-threoninol-conjugated TEMPO. (a-c) The MALDI-TOF-MS analyses of DNA oligonucleotides 5'-GGTATCTeGCAATC-3' synthesized using the amidite monomer of D-thr-TEMPO using a) the standard protocol for solid-phase DNA synthesis, b) with modification of the detritylation step using dichloroacetic acid, or c) with modification of the oxidation step using *tert*-butyl hydroperoxide. The crude products were analyzed by MALDI-TOF-MS after Polypack purification. Red arrows show target product and black arrows show DNA with reduced TEMPO moieties. (d) MALDI-TOF-MS analysis of RNA oligonucleotide 5'-GCAUCTeAGUC-3'. The RNA containing D-thr was synthesized using standard solid-phase synthesis protocols and 4-carboxy TEMPO was conjugated to the oligonucleotide post-synthetically.

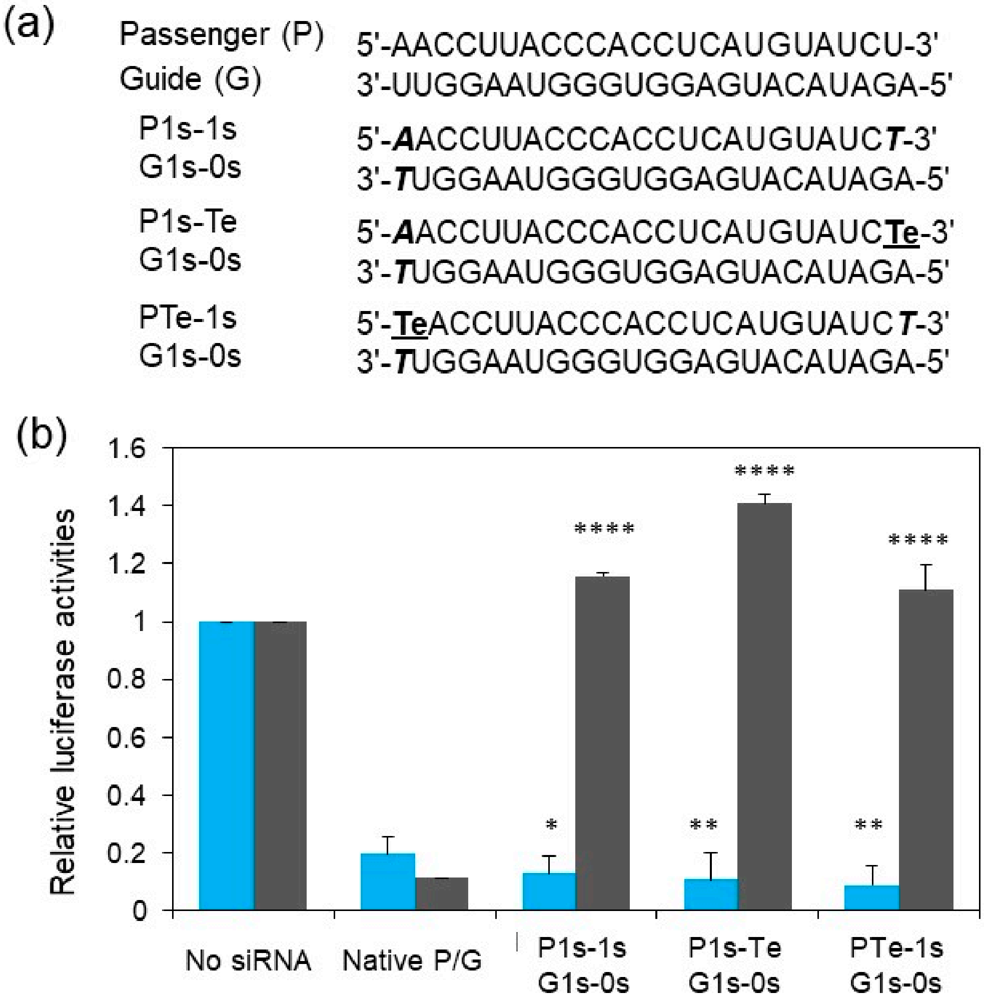

Figure S3. RNAi activities of TEMPO- and SNA-modified siRNAs. (a) siRNAs used in the luciferase assay. Te indicates the TEMPO conjugated through the D-threoninol backbone. Bold, italic characters indicate SNA. p indicates 5' phosphate. (b) RNAi activities of the guide strands (cyan bar) and passenger strand (grey bars) evaluated using reporter plasmids pGlo-On and pGlo-Off. Expression vectors and indicated siRNA (16.7 nM) were co-transfected into 293FT cells. After 48 h of incubation, luciferase activity was determined. Plotted are means and SD ($n = 3$) of firefly to *Renilla* luciferase activity relative to the untreated sample. *$p < 0.05$, **$p < 0.01$, ***$p < 0.001$, and ****$p < 0.0001$ vs. native P/G.

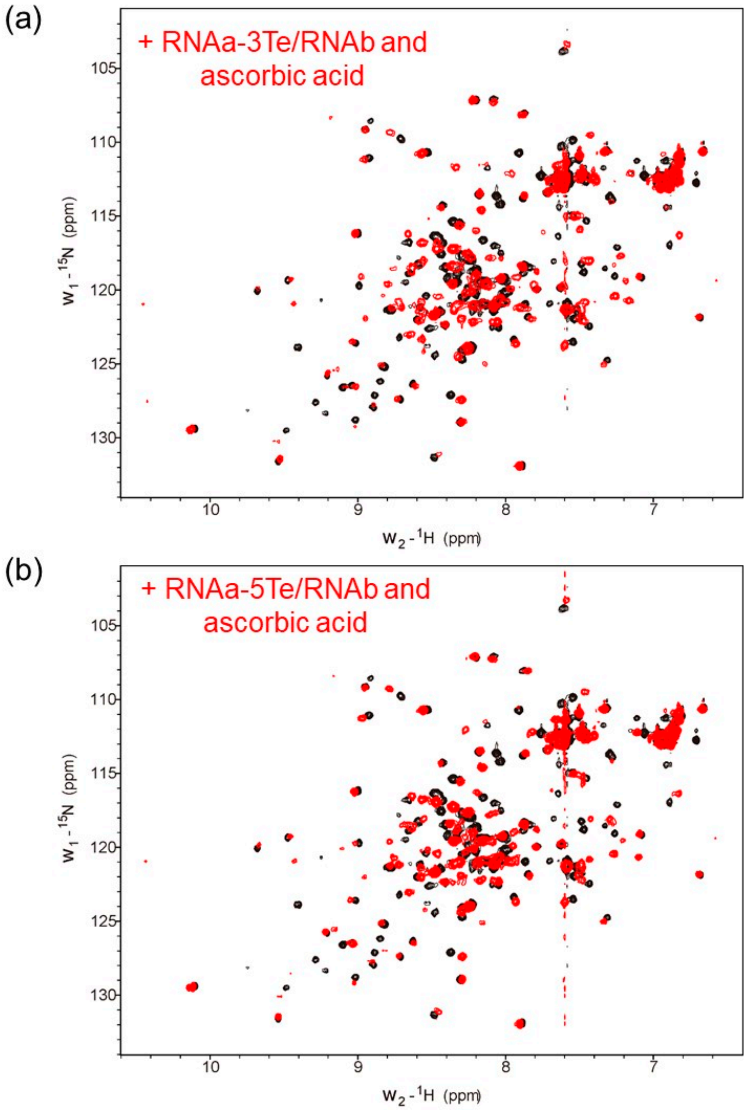

Figure S4 ^{1}H-^{15}N HSQC spectra of AGO2-MID complexed with RNA. ^{1}H-^{15}N HSQC spectra of AGO2-MID complexed with a) RNAa-3Te/RNAb and b) RNAa-5Te/RNAb in the presence of ascorbic acid (red) superimposed on the spectra of AGO2-MID (black). Samples contained 0.06 mM ^{15}N-labeled AGO2-MID and 0.4 mM RNA and 2 mM ascorbic acid to quench the TEMPO. NMR experiments were performed at 293 K in 25 mM MES (pH 6.5), 100 mM NaCl, and 10% (v/v) D_2O.

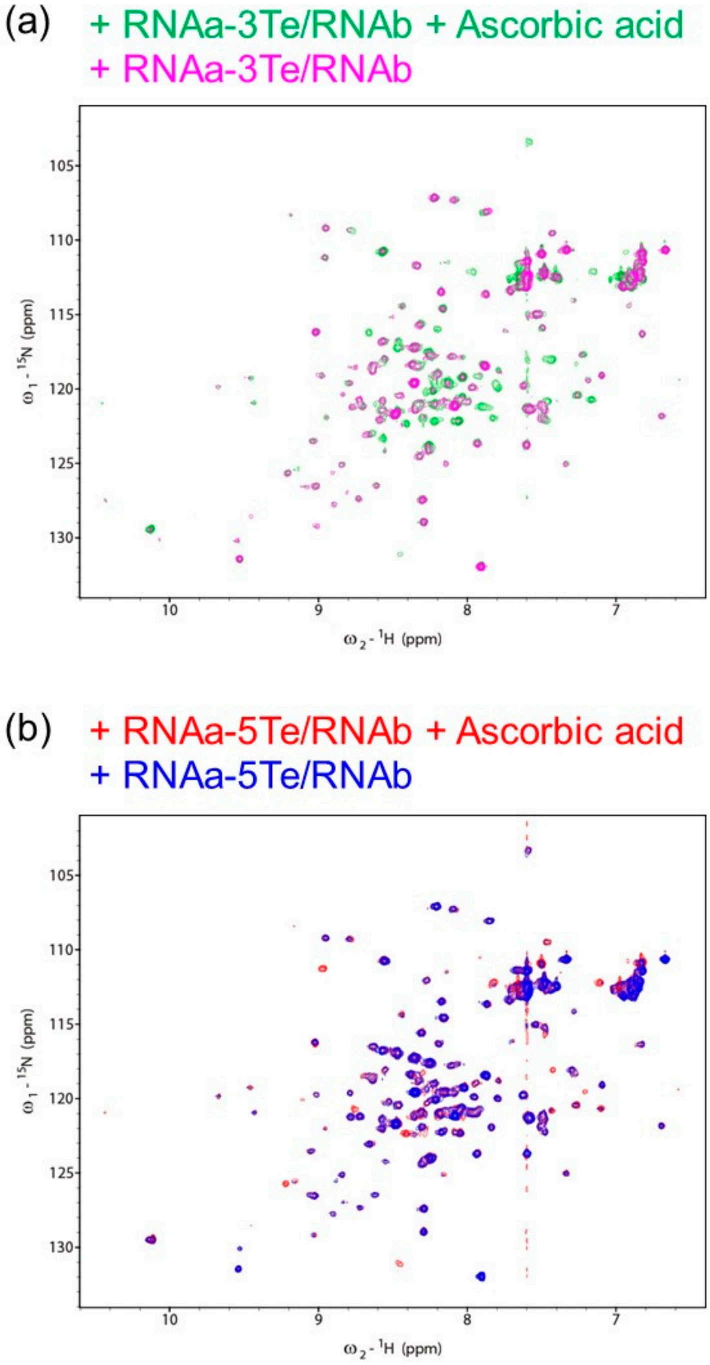

Figure S5 ^{1}H-^{15}N HSQC spectra of AGO2-MID complexed with TEMPO-RNA in the absence or presence of ascorbic acid. ^{1}H-^{15}N HSQC spectra of AGO2-MID complexed with a) RNAa-3Te/RNAb in the presence of ascorbic acid (green) or absence (magenta) of ascorbic acid or b) RNAa-5Te/RNAb in the presence (red) or absence (blue) of ascorbic acid. Samples contained 0.06 mM ^{15}N-labeled AGO2-MID and 0.4 mM RNA with or without 2 mM ascorbic acid and 25 mM MES (pH 6.5), 100 mM NaCl, and 10% (v/v) D_2O.
