# Supplementary figures and images for: Investigation of Strand-Selective Interaction of SNA-Modified siRNA with AGO2-MID
Source: Int J Mol Sci. 2020 Jul 23;21(15):5218. doi: 10.3390/ijms21155218 (PMC7432901; doi:10.3390/ijms21155218)

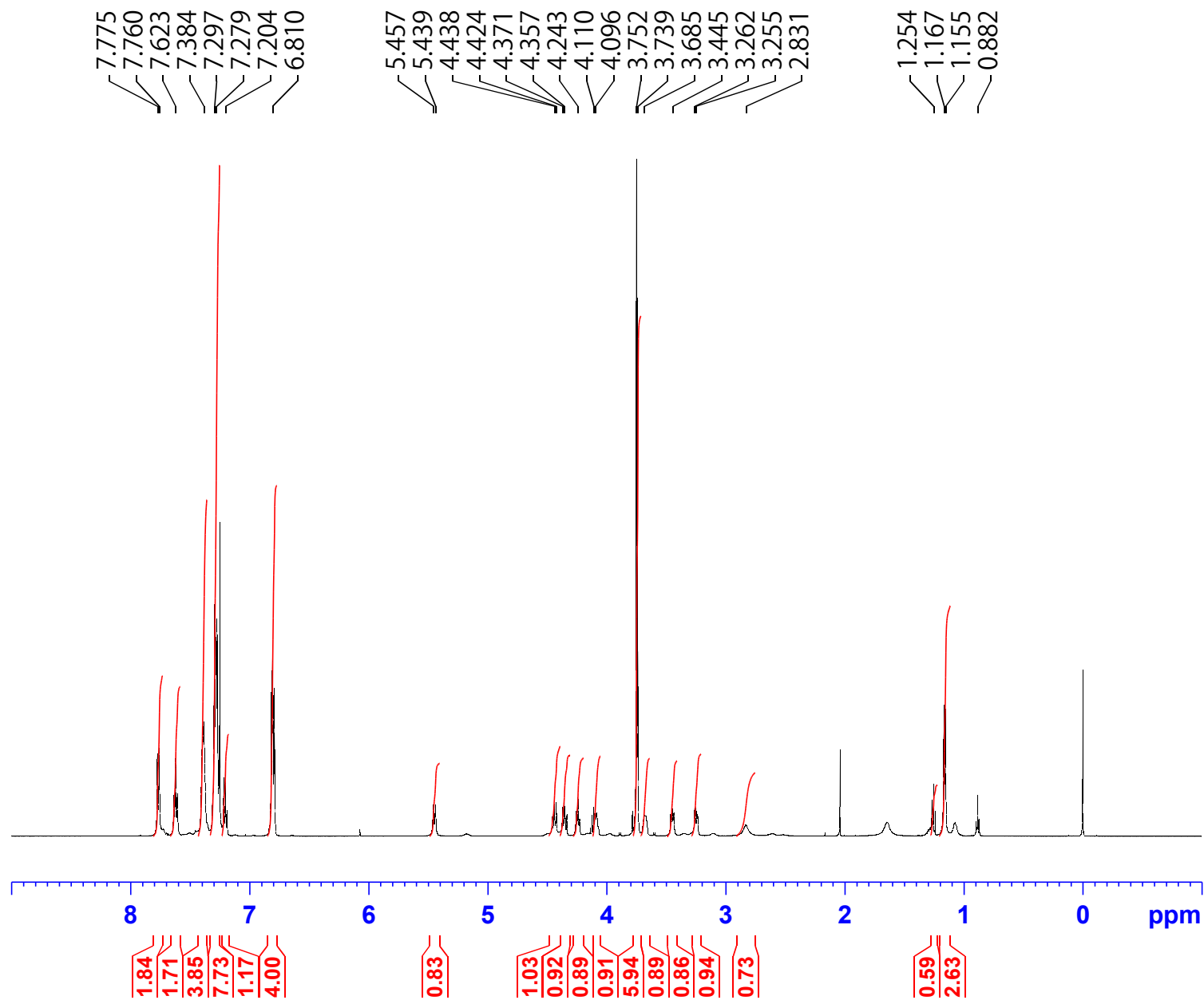

**<sup>13</sup>C-NMR of  
compound 3**

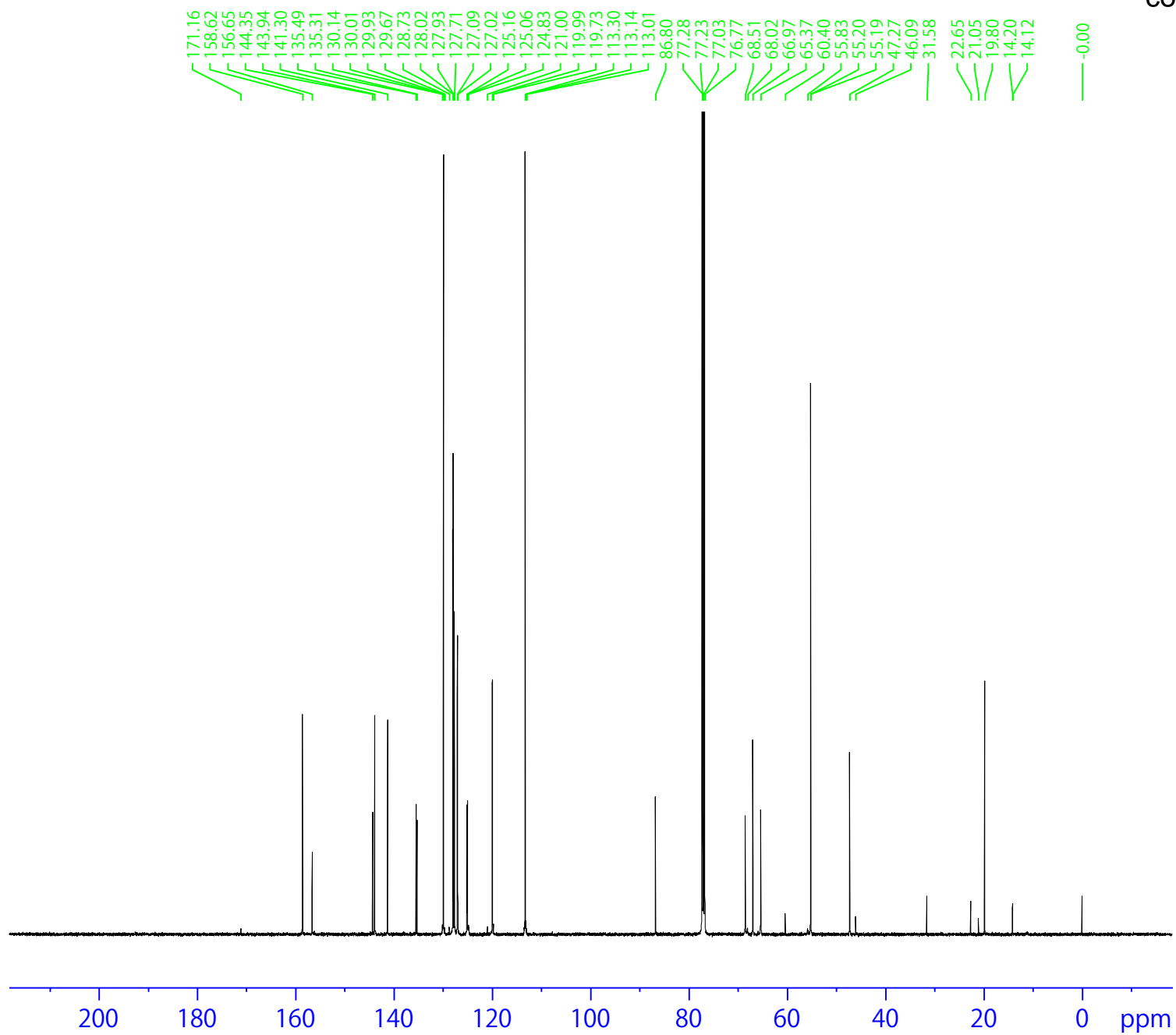

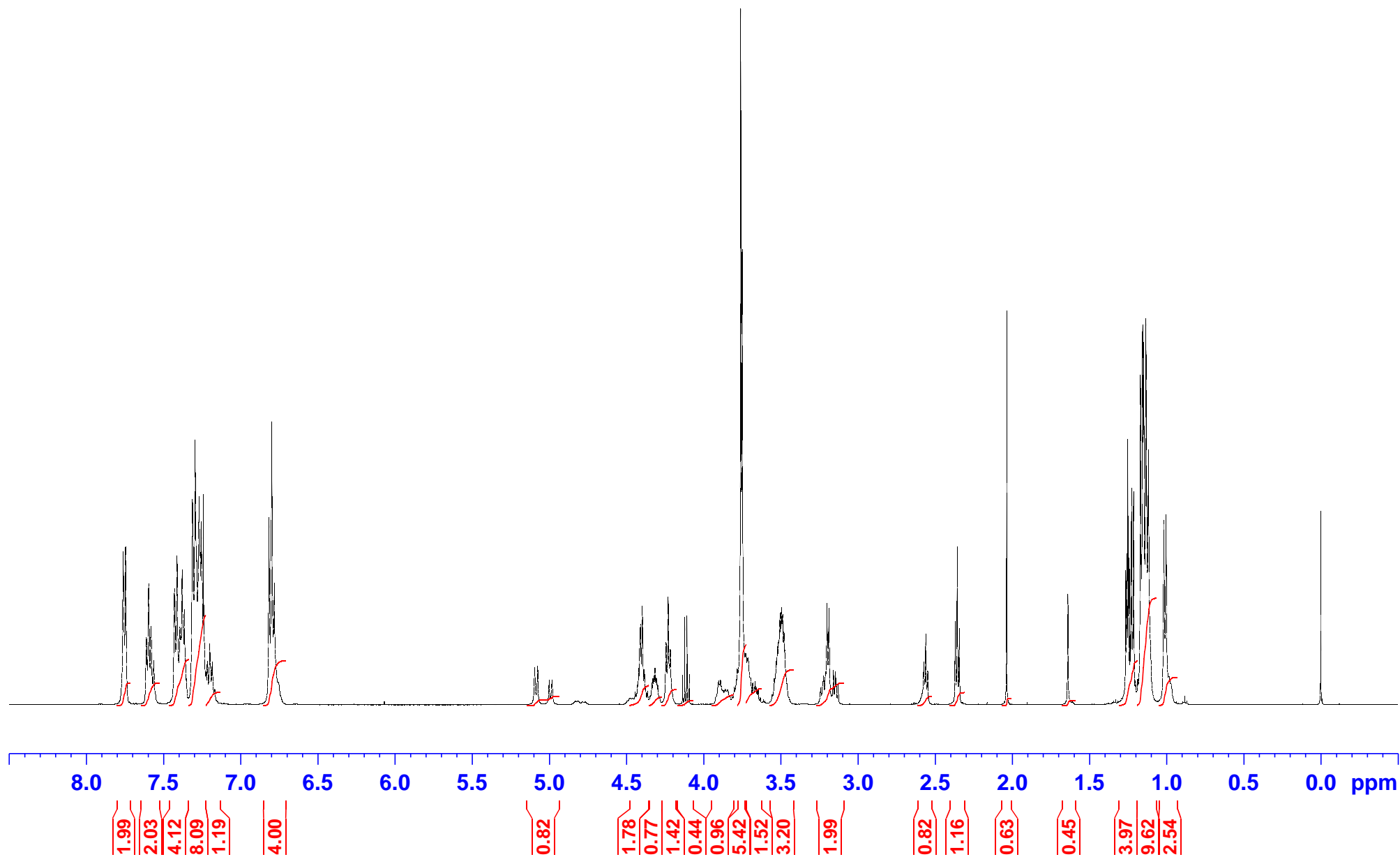

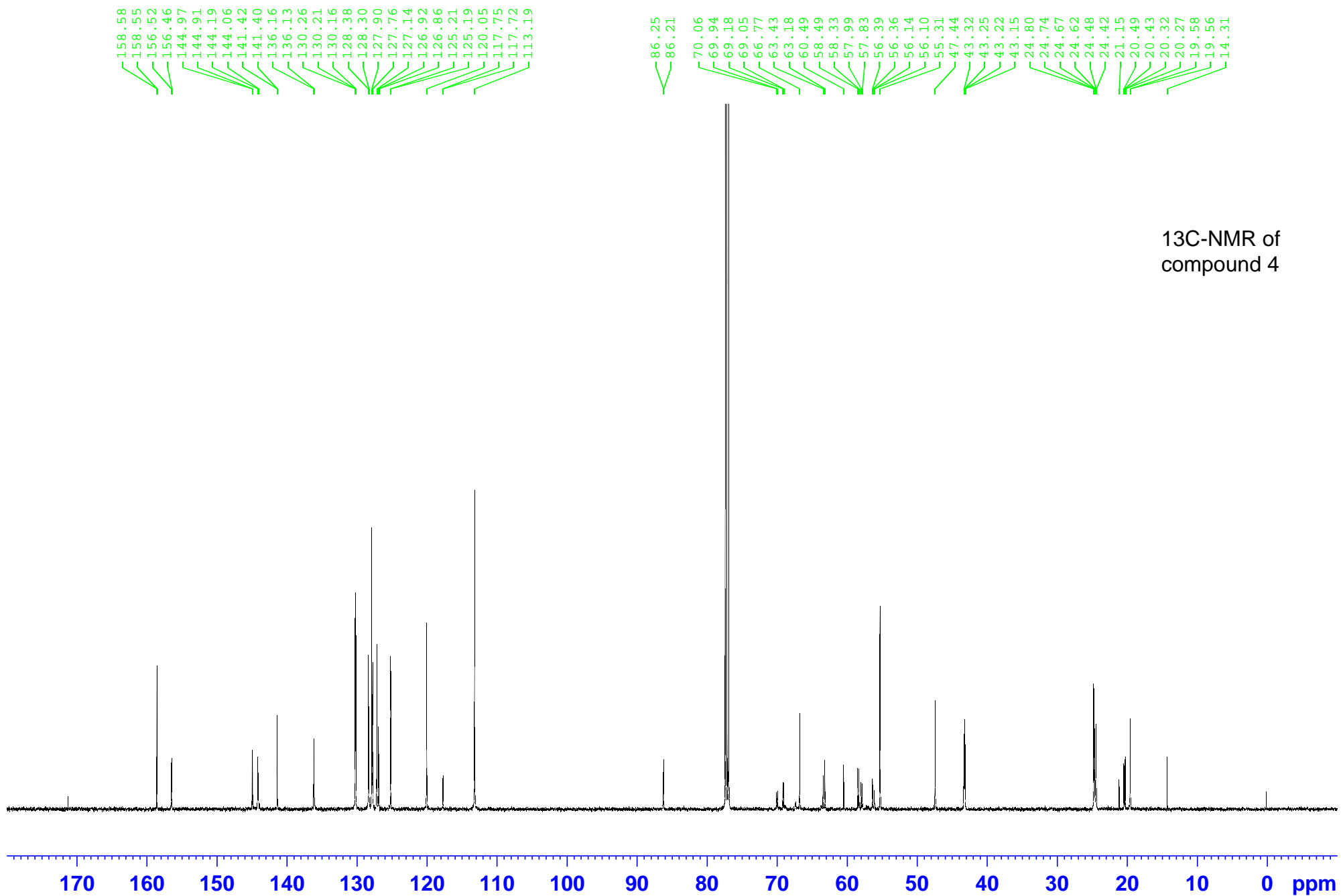

<sup>13</sup>C-NMR of  
compound 4

31P-NMR of  
compound 4

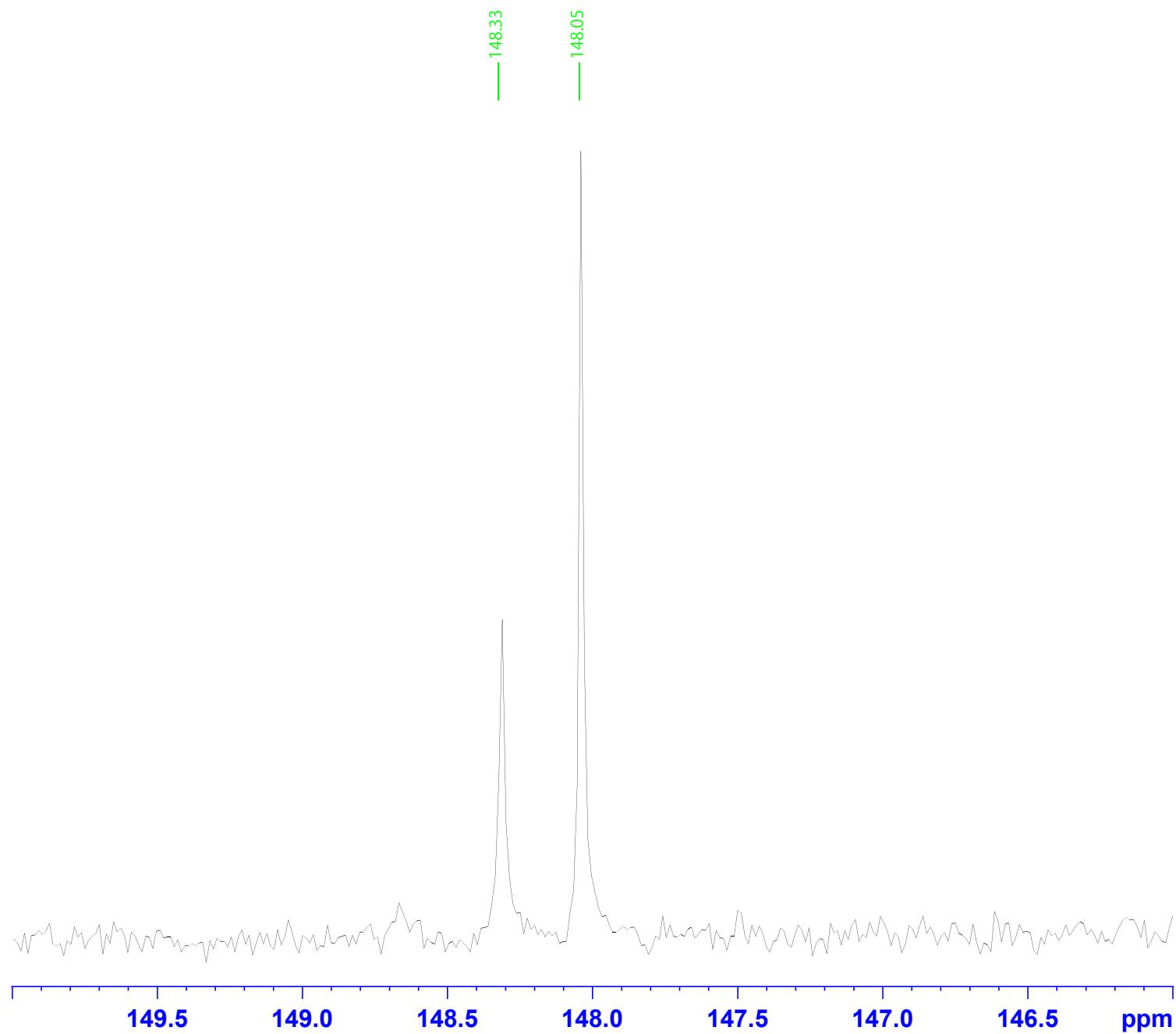

Supplement: Supplementary file 1 [file ijms-21-05218-s001.zip › TEMPO-RNA_Supporting_proof_S9-S13.pdf]
